# The impact of basic dermatology education and training on primary healthcare providers in KwaZulu-Natal, South Africa

**DOI:** 10.4102/safp.v63i1.5200

**Published:** 2021-01-15

**Authors:** Pumeza U. Makaula, Antoinette V. Chateau, Richard J. Hift, Ncoza C. Dlova, Anisa Mosam

**Affiliations:** 1 Department of Dermatology, School of Clinical Medicine, University of KwaZulu-Natal, Durban, South Africa; 2 Department of Medicine, School of Clinical Medicine, University of KwaZulu-Natal, Durban, South Africa

**Keywords:** dermatology education, dermatology training, South Africa, primary healthcare, common dermatology conditions

## Abstract

**Background:**

Dermatological diseases are amongst the commonest reasons for consultation at primary care level. Yet, dermatology teaching in medical and nursing curricula is inconsistent and often insufficient to enable medical and nursing professionals to manage these conditions effectively.

**Methods:**

We tested the knowledge of 100 doctors and 195 nurses who attended dermatology training sessions held in three health districts in the province of KwaZulu-Natal (KZN), South Africa, by using a quasi-experimental uncontrolled before-and-after study design. At the start of the session, participants were exposed to 15 slides representing common dermatological conditions; this was followed by a test. The participants then attended a series of short lectures followed by the same test. Pre- and post-intervention test scores were compared, and the results were analysed by professional status, health district and type of facility.

**Results:**

The mean (standard deviation [SD]) pre-intervention test score was 40.6% (20.5%). Doctors scored significantly higher than nurses (*p* < 0.0001). There were significant differences in performance by district (*p* < 0.001) and type of facility (*p* < 0.001). The mean (SD) post-intervention score improved to 68.7% (22.5%).

**Conclusion:**

Doctors and nurses working in the primary care sector appear to be insufficiently trained in the management of common dermatological conditions. A short period of in-service training resulted in an immediate, significant improvement in knowledge, although we did not study long-term retention beyond this. We recommend improved prequalification training in dermatology in medical and nursing schools and an expansion of continuing professional development as well as in-service training opportunities for primary care practitioners.

## Introduction

More than 1000 skin or skin-related conditions are listed by the International Classification of Disease 10 (ICD 10), and skin disease affects 30% – 70% of the global population.^1^ It has been estimated that between 6% and 24% of all primary care consultations are skin related, making dermatological conditions one of the commonest reasons for consultation.^2,3^ Yet, it has been suggested that insufficient attention is paid to skin conditions in the national and global health discourse.^4^ Pyoderma, scabies and superficial mycoses have a high prevalence in the general population, particularly in children,^5,6^ and expenditure on these conditions is relatively high,^4,7^ yet medical and nursing professionals may be poorly equipped to manage these dermatoses.^8^ The high costs associated with these disorders may in part be because of a lack of proficiency amongst primary care practitioners, who may lack the knowledge to diagnose and treat these conditions appropriately.^9,10^ The high incidence of human immunodeficiency virus (HIV) infection in KwaZulu-Natal (KZN), where seroprevalence increased from 15.5% in 2014 to 27% in 2018,^11,12^ has resulted in an increase in the number of patients with HIV-related skin conditions presenting to primary healthcare centres (PHCs).^13^ Dermatological conditions have both a physical and psychological impact on patients.^14^ Patients with disfiguring skin conditions are susceptible to psychological distress,^15,16^ and those with acne, psoriasis and atopic dermatitis are prone to reactive depression and suicide.^15,16,17,18^ Recognition of this has led to the development of the field of psychodermatology.^19^ Adequate care of patients therefore requires a holistic biopsychosocial approach.^20^ If this is to be successful, it is critical that clinicians are knowledgeable about common dermatological conditions and proficient in the recognition and management thereof.

As of October 2019, there were 265 dermatologists registered by the Health Professions Council of South Africa (personal communication, Health Professions Council of South Africa). A total of 42 are based in KZN, with only 12 working in the public sector, serving a population of over 11 million (Prof. Anisa Mosam, personal communication). The uninsured population is therefore grossly underserved by dermatologists, and the vast majority of patients presenting with skin conditions are cared for by non-specialist medical and nursing professionals working at a primary care level.

In this study we set out to assess the level of knowledge of recognition, diagnosis and management of common skin conditions amongst medical and nursing professionals working in the primary care setting in three urban centres in KZN and to assess the short-term effect of a structured training session designed to improve such knowledge.

## Methods

### Study design

We used a quasi-experimental uncontrolled before-and-after study design.

### Participants

All nursing and medical staff working at primary healthcare clinics, community health centres and district hospitals in three health districts were invited via the Department of Health office to attend a free single training session offered in each of the three districts. Clinics, community health centres and district hospitals perform defined roles within a hierarchy of care within the primary care tier of the South African public healthcare system; management is essentially nurse-led in the clinic, nurse- or doctor-led in the community health centre and doctor-led in the district hospital. Problems of increasing complexity are referred upwards for diagnosis and management within this hierarchy, and in this study we distinguish them as ‘type of facility’. Potential participants were informed that the study would be conducted at the training sessions and that they would be invited to participate.

### Study site

The study was conducted in three urban centres in the KZN province: eThekwini Municipality (greater Durban), Pietermaritzburg and Stanger. We chose these because they are the principal towns in the eThekwini, Umgungundlovu and Ilembe health districts, respectively. A single study session was held in each of the three centres, and the centres were studied over 3 consecutive days.

### Study procedures

**FIGURE 1:**
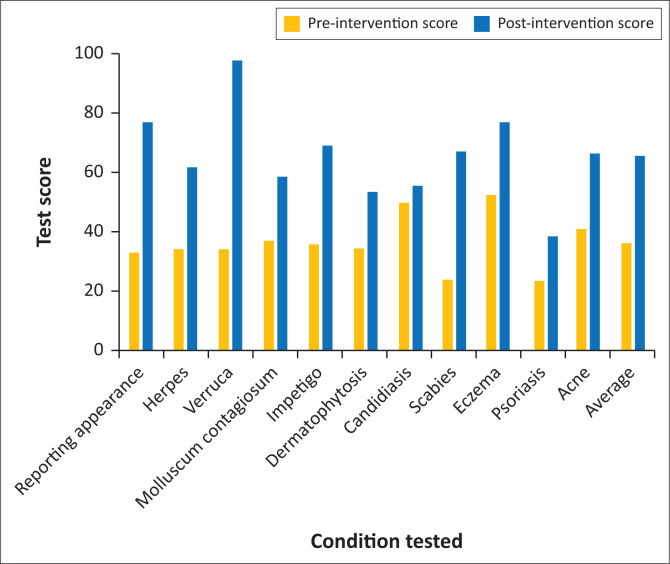
Breakdown of average performance by each of the 11 topics.

A total of 295 participants were subjected to a short pre-intervention test. They were exposed to 15 projected colour slides, representing common dermatological conditions, with 1 min allocated per slide. Each slide was accompanied by a variable number of questions covering the diagnosis, treatment and complications of the illustrated condition, provided to the participants in a questionnaire, on which they provided written answers. The 15 slides covered 11 topics (Figure 1). One mark was awarded for each unambiguously correct answer. There were no partial marks. The total possible score was 40.

The participants then attended a series of 15-min lectures given by consultant dermatologists working in the dermatology departments of KZN public-sector hospitals. Ten topics were presented, covering the clinical description of skin lesions, viral, bacterial, parasitic and fungal infections, eczema, acne, psoriasis, vitiligo, disorders of pigmentation, dermatological side-effects of skin bleaching products, HIV-related skin diseases and knowledge of which conditions were appropriate for referral for specialist assistance. The total duration of the training session was 150 min. The lectures included all the knowledge potentially required to achieve full marks on the test. Following the lectures, the participants retook the same test, which they had attempted earlier, under the same conditions. The total duration for both assessments was 30 min.

### Data analysis

Data were captured on a password-protected Microsoft Excel spreadsheet and analysed by using MedCalc Statistical Software version 19.1.5 (MedCalc Software, Belgium; https://www.medcalc.org; 2020). Pre- and post-intervention test scores were compared by using Student’s *t*-test for paired samples. Scores on the two tests were compared between the three sites with one-way analysis of variance (ANOVA) and post hoc with the Tukey–Kramer test. The improvement ratio was compared between the three sites with the Kruskal–Wallis test and post hoc with the Conover test. Interactions between profession, site and type of facility were tested with two-way factorial ANOVA. A *p*-value less than 0.05 was considered significant.

### Ethical consideration

The research study was approved by the Biomedical Research Ethics Committee of the University of KwaZulu-Natal (reference number: BFC314/17). All participants provided oral informed consent. Participation was anonymised; unique codes were assigned to each participant allowing the reconciliation of pre- and post-test results.

## Results

We studied 295 participants: 195 nurses and 100 doctors. The mean (standard deviation [SD]) age for all participants was 41.1 (10.5) years. The mean (SD) age for nurses was 42.3 (10.5) years and for doctors 38.7 (10.1) years. The difference is significant (*p* = 0.005). There were no significant difference in the ages of staff at the different centres (*p* = 0.38), or by type of facility (*p* = 0.58) (data not shown). Test performance is summarised in Table 1 and performance by topic shown in Figure 1.

**TABLE 1:** Performance on the test measured before and after a teaching intervention.

Participants tested	Pre-intervention		Post-intervention		Significance	Improvement†	
	Mean	SD	Mean	SD		Pre-intervention (median)	Post-intervention (IQR)
All participants (*n* = 295)	40.6	20.5	68.7	22.5	*p* < 0.0001	1.7	1.2–2.8
**By profession**							
All doctors (*n* = 100)	57.5	16.5	71.6	67.2	*p* < 0.0001	1.3	1.0–1.4
All nurses (*n* = 195)	31.9	16.6	67.2	21.6	*p* < 0.0001	2.2	1.43–3.75
**By district**							
eThekwini (*n* = 101)	49.7	20.8	76.5	23.3	*p* < 0.0001	1.5	0.1–2.3
uMgungundlovu (*n* = 92)	35.5	15.6	59.3	19.1	*p* < 0.0001	1.6	1.1–2.6
Ilembe (*n* = 102)	36.2	21.4	69.4	21.5	*p* < 0.0001	1.9	1.3–3.8
**By type of facility**							
Clinic (*n* = 133)	34.5	17.3	65.2	21.5	*p* < 0.0001	1.9	1.2–3.5
Community health centre (*n* = 59)	40.0	18.5	74.4	22.3	*p* < 0.0001	1.9	1.4–2.5
District hospital (*n* = 103)	48.8	22.7	70.0	23.2	*p* < 0.0001	1.4	1.1–2.1

Note: The scores are expressed as a percentage and are shown as mean (SD).

IQR, interquartile range; SD, standard deviation.

† , Improvement is expressed as the ratio of post-intervention score to pre-intervention score and is shown as median (interquartile range).

Performance in the pre-intervention test was poor, with a mean (SD) score of 40.6% (20.5%). Medical professionals scored significantly higher than nursing professionals (*p* < 0.0001). There were significant differences in performance by district (*p* < 0.001) and type of facility (*p* < 0.001), with the highest scores noted in district hospitals and in the eThekwini Health District.

**FIGURE 2:**
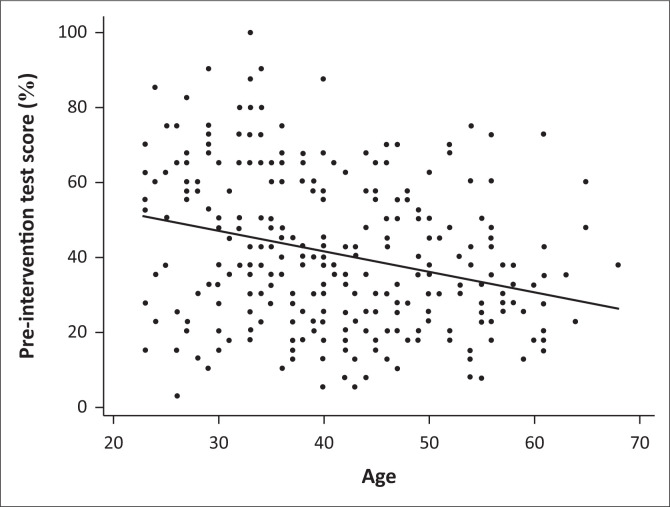
Correlation between age and pre-intervention test score.

Given the differential performance between medical and nursing professionals, we considered the possibility that the differences between districts and type of facility might have been confounded by differences in the ratios of doctors to nurses within these. Two-way factorial ANOVA excluded a significant interaction between profession and both district (*p* = 0.33) and type of facility (*p* = 0.15), suggesting that the observed differences indicate real differences in performance. There appeared, however, to be some interaction between district and type of facility (*p* = 0.04). There was a significant negative correlation between age and pre-intervention score (*r* = -0.28, confidence interval [CI] -0.38–0.17, *p* = 0.001; Figure 2). This was shown for both medical professionals (*r* = -0.32, CI -0.49–0.13, *p* = 0.001) and nursing professionals (*r* = -0.19, CI -0.32–0.05, *p* = 0.009).

**FIGURE 3:**
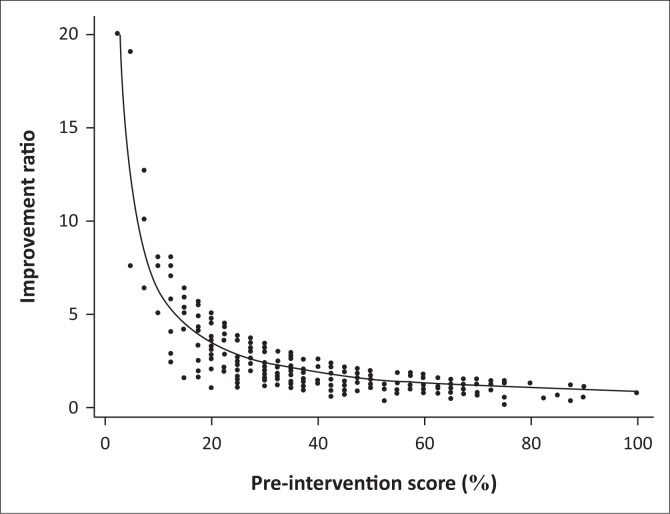
Improvement in scores is inversely proportional to the pre-intervention test score, with those who scored poorly initially improving by a proportionately greater degree than those with initially higher scores. The improvement ratio is calculated as the post-intervention score divided by the pre-intervention score.

The mean (SD) post-intervention score was 68.7% (22.5%), indicating a significant improvement over pre-intervention scores (*p* < 0.0001). Post-intervention scores were 1.7 times higher than the pre-intervention scores. This improvement was noted for both medical and nursing professionals and for all districts and type of facility. Participants who scored poorly on the pre-intervention test improved proportionately more in the post-intervention test than those with higher initial scores (Spearman’s rho -0.81, CI -0.85–0.77, *p* < 0.0001, Figure 3), suggesting a significant catch-up in performance. There was no significant difference in the post-intervention scores of medical and nursing professionals (*p* = 0.25), although significant differences persisted between the three districts (*p* < 0.001) and between the three types of facilities (*p* = 0.03).

## Discussion

Most public-sector patients seeking assistance with a dermatological problem experience their first interaction with the health service in a primary care setting. The low scores obtained on the pre-intervention test in our study suggest that most clinicians working in primary care in the three districts we tested are not adequately prepared to manage common skin conditions. This might potentially result in incorrect diagnosis and treatment and a higher incidence of complications of simple skin conditions. Knowledge appeared to be weak across a range of common skin conditions. Even topics that at face value appear simple and frequently encountered, for example scabies, were answered no better than those which might be regarded as more complex, such as psoriasis.

Skin disorders are common in the community and therefore frequently encountered by primary care practitioners. Our results however suggested that this experience has not led to adequate proficiency in diagnosis and management. The significant negative correlation between proficiency and age we identified may suggest that knowledge decay with time is more relevant than increasing experience in determining proficiency. Alternatively, it may reflect better undergraduate teaching amongst more recent graduates. In both cases, however, the overall poor proficiency indicates either a deficiency in undergraduate training or a continuous inadequate in-service professional development in the field of dermatology during clinical practice or both. Our own enquiries suggest that formal exposure to dermatology training across South African medical schools may range from as little as 15 to 240 h, spread across a 5-or 6-year curriculum.

Given the poor knowledge of common skin disorders that we have shown in this study, we suggest that the general level of dermatological expertise amongst nurses and doctors working in primary care should be improved. This is likely to require a multifaceted approach, including improved dermatology training in nursing colleges and medical schools, repeated exposure to continuing professional development programmes and in-service training and improved communication and collaboration between primary care practitioners and specialist dermatologists. There are also very limited opportunities for diploma studies in dermatology practice for primary care practitioners.^21^

Our study has shown that even a brief intervention, not requiring more than 3 h on a single day, is sufficient to bring about a substantial increase in immediate knowledge, as shown previously in both KZN^22^ and Mali.^23^ Our study looked only at a short-term improvement in test performance, but not at retention of improvement or improvement in practice. We recognise that without reinforcement, the degree of improvement shown may well not be maintained indefinitely, thus requiring regular and recurring exposure to dermatology training opportunities.

### Study limitations

The post-intervention test was carried out immediately after the intervention effectively controls for a weakness of before-and-after studies, in that the effect of other possible influences, such as temporal trends in knowledge or exposure to other factors, has been negated.^24^ Against this, however, the immediacy of the post-intervention testing does not allow us to evaluate the retention of knowledge, deeper understanding and its effectiveness in improving subsequent practice.

We did not apply a standard format for questioning within each topic; questions for some topics may have been weighted towards diagnosis and others to treatment. This does not allow us to identify rigorously which areas of knowledge are weakest and require the most attention.

### Conclusion and recommendations

We conclude that the knowledge of skin disorders amongst doctors and nurses working in the state primary care sector is inadequate. Addressing this may require a number of interventions in parallel, including curricular change in nursing and medical schools, improved continuing professional development and in-service training in skin disorders for primary care practitioners and extensional diploma programmes for qualified practitioners. In this study we have shown that a short-term educational intervention results in a significant and immediate increase in knowledge. We recognise, however, that an ongoing engagement is necessary to ensure that such improvement is maintained.
